# Intervention thresholds: a conceptual frame for advance care planning choices

**DOI:** 10.1186/1472-684X-13-21

**Published:** 2014-04-10

**Authors:** Karen G Scandrett, Brian Joyce, Linda Emanuel

**Affiliations:** 1 Northwestern University, Buehler Center on Aging, Health & Society, Chicago, Illinois, USA; 2 General Internal Medicine, 750 N. Lake Shore Drive, Suite 601, Chicago 60611, Illinois, USA

**Keywords:** End-of-life, Advance care planning, Decision making

## Abstract

**Background:**

Advance care planning (ACP) provides for decisions in the event of decisional incapacity. Determining ahead of time what a person may want is challenging and limits the utility of ACP. We present empirical evidence for a new approach to ACP: the individual’s “intervention threshold.” The intervention threshold is intuitively understood by clinicians and lay people, but has not been thoroughly described, measured, or analyzed.

**Methods:**

Using a mixed-methods approach to address the concept of the intervention thresholds, we recruited 52 subjects from a population of chronically ill outpatients for structured telephone interviews assessing knowledge, attitudes, and prior ACP activities. Respondents were presented with 11 interventions for each of four medical scenarios. For each scenario, they were asked whether they would accept each intervention. Data was evaluated by descriptive statistics and chi-squared statistics.

**Results:**

Complete data were obtained from 52 patients, mean age of 64.5, 34.6% of whom were male. Only 17.3% reported prior ACP discussion with a physician. Rates of accepting and refusing interventions varied by scenario (p < 0.0001) and intervention intensity (p < 0.0001).

**Conclusions:**

These data provide evidence that people display transitions between wanting or not wanting interventions based on scenarios. Further research is needed to determine effective ways to identify, measure, and represent the components of an individual’s intervention threshold in order to facilitate informed decision making during future incapacity.

## Background

For many patients, physical or cognitive decline accompanying severe illness makes them unable to choose medical intervention according to their wishes. This is primarily addressed through advance care planning (ACP), [1] Despite some successes [2,3] ACP is underutilized [4] and adherence can be difficult [5,6]. Unstructured or semi-structured living wills (i.e., Five Wishes) [7] can be difficult to interpret, predetermined physician orders (i.e., POLST) may not always apply, and proxies may lack knowledge [8] or worry that prior wishes may not represent present realities, thereby experiencing considerable burden [9]. ACP communication must be improved so that treatments align with patients’ goals [10] and surrogates are adequately prepared for future decision-making [11,12]. Some newer ACP approaches, which have improved directive completion, include software-guided discussion [13] and facilitated discussion by members of the healthcare team such as social workers or clergy [14,15].

Nonetheless, it remains difficult to reliably identify patient wishes regarding care decisions. We propose that the concept of intervention thresholds may help insofar as it has intuitive appeal and potential widespread utility. Many clinicians perceive that patients reach a threshold at which they feel treatment burden outweighs its possible benefit, whereupon curative interventions that risk pain, discomfort or worsened functional status are declined and the focus shifts toward other important tasks of the dying role [16]. This concept has been explored in the scientific literature [17]. One group used a decision-analytic approach to discern evolving treatment preferences across a shifting probability of outcomes, and demonstrated that the approach produced internally consistent, reliable results [18]. However, thresholds have not been systematically investigated as an organizing theme for ACP nor operationalized for clinical use. This study presents evidence that individual thresholds may be ascertained using straightforward, illustrative clinical examples. Our goals in this descriptive study are to solidify grounds for investigating this approach and to generate hypotheses for future inquiry.

## Methods

We obtained Institutional Review Board approval for this research prior to study activities (Northwestern University IRB # STU00030974). We collected information using an instrument designed to elicit care preferences across the spectrum of clinical situations in which decisional capacity is impaired [18,19]. The instrument was previously examined for validity and shown to correlate specific preferences with general goals of care [20,21]. Our objective was to determine whether the *transition* from primarily curative to palliative goals was ascertainable using specific intervention choices.

For this formative study we sought to sample a population of older adults with chronic medical illness. Eligible subjects had been seen 2–4 times at a general internal medicine clinic of a large urban medical center in the preceding six months, had more than three chronic medical conditions documented in their medical record, were age 18+, and spoke English. Randomly generated lists of potential subjects were provided to investigators, who contacted the primary care physician to request exclusion of anyone they felt would be unwilling or unable to participate. Eligible subjects were invited to participate via letter and one follow-up phone call within five days.

After consenting, subjects participated in a 20-minute phone interview. They reported their overall health on a scale of 1 (poor) to 4 (excellent), number of hospitalizations in the past year, and whether they thought their conditions would shorten their life. Prior ACP was queried (e.g., completing an advance directive or power of attorney for healthcare, or discussing ACP with their doctor). Four scenarios were presented, representing degrees of acuity (Table 1), and for each scenario subjects were asked whether they would accept, decline, or were undecided about each of eleven interventions. Final questions collected demographic information including age, sex, race/ethnicity, and religion. Unsolicited comments were recorded in field notes.

**Table 1:** Health scenarios presented to subjects via telephone interview

**Scenario description**	
Scenario 1:	You are in a coma with a small likelihood of recovering fully, a slightly larger likelihood of surviving with permanent brain damage, and much larger likelihood of dying.
Scenario 2:	You are in a coma/persistent vegetative state, with no hope of regaining awareness and higher mental functions no matter what is done.
Scenario 3:	You have brain damage or disease which cannot be reversed and makes you unable to recognize people or to speak understandably, and a terminal illness which will likely be the cause of your death.
Scenario 4:	You have brain damage or disease which cannot be reversed and makes you unable to recognize people or to speak understandably, without any other condition that is likely to cause your death, and you could live in your current condition for a long time.

Scenarios were derived and validated for an Advance Care Planning Document [19].

After transcription, subjects who provided the same Yes or No response to more than 90% of the 44 scenario-intervention pairs were considered to either have a threshold not covered by the scenarios or no threshold at all. Yes/No/Undecided data from those remaining were graphed using Microsoft Excel to identify a transition in care choices. Subjects were determined to have crossed their threshold when they changed from accepting to refusing most interventions from one scenario to the next. Interventions were then grouped based on invasiveness, burden, cost, and duration, resulting in a categorical variable representing intervention intensity. The three levels of intensity were defined by the investigators as follows: Non-intensive interventions (Simple Diagnostic Tests, Antibiotics); minimally intensive interventions (Artificial Nutrition/Hydration, Minor Surgery, Invasive Diagnostic Tests, Blood/Blood Product Transfusions); and intensive interventions (CPR, Mechanical Breathing, Major Surgery, Hemodialysis, Chemotherapy). For statistical analysis, we excluded all ‘undecided’ responses and used extensions of the Fisher’s exact test (SAS version 9.2) to test whether the distribution of Yes/No responses varied (one test for variation by scenario and another by intervention intensity). Our threshold for statistical significance was set at α = 0.05. Field notes were also reviewed to provide further information about subject thought processes.

## Results

For this exploratory, hypothesis-generating study a convenience sample of 52 subjects from a total population of 556 individuals invited participated in the full interview (Table 2). Our sample was 69.2% white, with a mean age of 64.5 (range 21–81 years) and 34.6% male. The majority (71%) of subjects rated their health as “Excellent” or “Good”. Only 17.3% reported having discussed ACP with their physician. Of all respondents, 57.7% reported having an advance directive, and 61.5% reported having a proxy.

**Table 2:** Subject characteristics (n=52)

**Subject characteristics (n=52)**	
Age	
≤ 50	7 (13%)
51-60	7 (13%)
61-70	16 (31%)
71-80	20 (39%)
80+	2 (4%)
Sex	
Male	18 (35%)
Female	34 (65%)
Race	
White/Caucasian	36 (69%)
Black/African-American	14 (27%)
Hispanic/Latino	1 (2%)
Asian-American	1 (2%)
Religion	
Catholic	19 (36%)
Protestant	18 (35%)
Jewish	4 (8%)
Muslim	1 (2%)
Agnostic	1 (2%)
Atheist	1 (2%)
Not specified	8 (15%)
Self-rated health	
Excellent	11 (21%)
Good	26 (50%)
Fair	14 (27%)
Poor	1 (2%)
Admitted to hospital in last year?	
Yes	29 (56%)
No	23 (44%)
Believed health conditions would shorten life?	
Yes	16 (31%)
No	29 (56%)
Don’t know/refused	7 (13%)
At risk for dementia?*	
Yes	15 (29%)
No	37 (71%)
Living will?	
Yes	34 (65%)
No	16 (31%)
Refused/not sure	2 (4%)
Power of attorney?	
Yes	34 (65%)
No	16 (31%)
Not sure	2 (4%)
Discussed with MD?	
Yes	10 (19%)
No	42 (81%)
% Declining CPR	
Scenario 1	40%
Scenario 2	71%
Scenario 3	75%
Scenario 4	77%

*Subjects were considered to be at risk for dementia if their medical history included one or more of the following conditions: Hypertension, any cardiovascular disease, diabetes, or high cholesterol.

Twenty-one (40%) subjects gave the same “no” or “yes” answer to at least 90% of all interventions. For this portion of our population, these scenarios did not capture a transition between accepting and declining intervention. Among these subjects, 17 (33%) declined at least 90% of all interventions, and the intervention requested was usually for simple diagnostics. For this group, their threshold could perhaps have been captured by a less dire scenario than those we used. For the 4 (8%) who accepted at least 90% of all interventions, a threshold of preference for non-curative care may have been identifiable with a particular scenario or may not exist.

Among the remaining 31 (60%) subjects, responses varied by clinical scenario (p < 0.0001) and intervention intensity (p < 0.0001). Within this subgroup, 21 (68%) crossed a threshold during the interview; 16 (52%) between scenarios 1 and 2, and 5 (16%) between scenarios 2 and 3. None of the respondents had a discernible threshold between scenarios 3 and 4. Within this subgroup thresholds were also ascertainable on the basis of intervention intensity, with 56% of subjects accepting non-intensive interventions, 38% accepting minimally intensive interventions, and 27% accepting intensive interventions (Table 3).

**Table 3:** Percentage of no/undecided/yes responses by scenario and by intervention intensity (N = 31)

**Percentage of no/undecided/yes responses by scenario (N = 31)**			
Scenario*	**Response**		
	**No**	**Undecided**	**Yes**
Scenario 1	21.70%	8.50%	69.79%
Scenario 2	67.74%	3.81%	28.45%
Scenario 3	73.90%	6.16%	19.94%
Scenario 4	70.67%	2.64%	26.69%
**Percentage of no/undecided/yes responses by intervention intensity (N = 31)**			
Intensity*	**Response**		
	**No**	**Undecided**	**Yes**
Non-intensive	39.52%	4.44%	56.05%
Minimally Intensive	55.85%	5.85%	38.31%
Intensive	68.23%	5.16%	26.61%

*p<0.0001 for distribution of Yes/No responses.

Field notes provided further insight. Several subjects indicated that hope for a full recovery influenced their decision to accept intensive interventions. Others reflected that treatment burden, including unanticipated side effects, factored into their threshold for intervention. Finally, some subjects considered the impact of the condition on loved ones, financial resources, personal values/beliefs, and strength of their coping mechanisms.

## Discussion

Our results provide empirical evidence that an approach using thresholds holds promise for ACP. This work corroborates anecdotal evidence, often discerned by clinicians, that approximate thresholds can be detected through the discussion of clinical scenarios. For this exploratory study of drivers of decision-making we used an existing, validated worksheet for advance care planning. We used a yes/no/undecided response format, with the assumption that the transition from yes to no denotes a threshold. However, two of the four proposed scenarios were conceptually similar and did not describe a clearly linear progression, which may have made it more difficult to identify a threshold between scenarios. Furthermore all the scenarios were somewhat dire, which likely precluded identification of subject with thresholds reached at a higher health/functional state. Nonetheless we did find significant numbers of subjects crossing the threshold as prognosis changed, and we identified a small number of subjects for whom no threshold for intervention was reached. The values underlying decision-making for these population subgroups warrant further investigation.

Our results suggest areas for further research. For example, unprompted comments implied that, consistent with prior literature, multiple dimensions are important to patients in end-of-life decision making including prognosis, disability level, and treatment burden (invasiveness and financial or other caregiver or patient burden) [22-24]. It is possible that these elements may be codified to allow more refined threshold assessment and guidance regarding health decisions. The optimal number of dimensions and the best way to detect and use them is still unclear.

A previous study by Fried, et al. [25] developed a quantitative measure of the threshold for treatment by using a range of probabilities for specific outcomes. Using scenarios, Winter [26] has examined the relationship between patient values and treatment interventions, and identified several values that strongly correlate with intervention preference. Our study adds to these results by determining that the presence of a threshold may be ascertainable using an intuitive format, laying groundwork for further work to determine relevant elements of thresholds and test the concept in a clinical setting. Rigorous, systematic collection of thresholds data will permit further exploration of their function, stability, and utility: how thresholds change, what causes such changes, and whether information gathered in advance proves useful for proxies.

### Strengths and limitations

This study has several limitations. This pilot study had small subject numbers. Our sample was skewed toward females who had previously engaged in advance care planning (65% with a living will or DPOA in place already) and therefore results may be biased toward those favoring less intensive medical care. Because of limited numbers we were unable to identify subgoups (e.g. by gender, educational level, religious identification) for whom intervention thresholds differed. In addition, our study took place in the US healthcare system; our framework may need adjustment in countries in which healthcare resources are distributed differently. Finally, medical decisions of individuals lacking medical knowledge are difficult to interpret. Subjects in this study were only given the study-prescribed longer definition of a scenario or intervention when specifically requested. However, we used a structured tool that has been validated across multiple settings and is stable over time. Moreover our interview format allowed us to gather additional qualitative information to develop hypotheses for further study.

## Conclusion

A robust method to characterize and measure thresholds may be an effective way to frame discussions about end-of-life values and revisit the discussion periodically, equipping clinicians to focus on scenarios or interventions provoking the greatest uncertainty in patients or families. Intervention thresholds may also equip proxy decision makers with knowledge of their patients’ core values when the patient has lost decisional capacity, resulting in less personal burden and greater ability to enact their role. The use of thresholds holds promise as a readily elucidated and applied approach to decision-making. Further exploration may define the dimensions of the intervention threshold, enabling creation of a user-friendly tool to guide specific decisions about the end-of-life.

## Competing interests

The authors declare that they have no competing interests.

## Authors’ contributions

KS designed the study, analyzed the data and wrote the manuscript. BJ conducted data collection, and assisted with both data analysis and manuscript preparation. LE designed the original survey instrument, supervised the study, and assisted with data analysis. All the authors approve this manuscript and agree to its submission.

## Pre-publication history

The pre-publication history for this paper can be accessed here:

http://www.biomedcentral.com/1472-684X/13/21/prepub
